# Zwischen Preisjägern, Datenschützern und Tech-Enthusiasten: Segmentierung des Virtual-Reality-Marktes am Beispiel Oculus

**DOI:** 10.1365/s40702-021-00817-w

**Published:** 2021-12-14

**Authors:** Julian Schuir, Ludger Pöhler, Frank Teuteberg

**Affiliations:** Unternehmensrechnung und Wirtschaftsinformatik, Universität Osnabrück, Osnabrück, Deutschland

**Keywords:** Virtual Reality, Conjoint-Analyse, Endverbraucherpräferenzen, Marktsegmente, Datenschutzbestimmungen, Virtual Reality, Conjoint Analysis, Consumer Preferences, Market Segments, Privacy Policies

## Abstract

Virtual Reality (VR) hat in den vergangenen Jahren erhebliche technologische Fortschritte verzeichnet und begonnen, sich im Endverbrauchermarkt zu etablieren. Insbesondere Facebooks Tochterunternehmen Oculus erzielte mit der Quest 2 hohe Absatzzahlen, wodurch das Produkt zum bisher meistverkauften VR-Headset avancierte. Gleichzeitig entfachte sich aufgrund Oculus neuer Datenschutzbestimmung, welche die Gerätenutzung an ein Facebook-Konto bindet, jedoch ein kontroverser Diskurs unter Datenschutzexperten. Endverbraucher stehen seither vor einem Dilemma. Sie müssen sich zwischen der Preisgabe sensibler Daten an Facebook im Falle der Nutzung kostengünstiger Oculus-Geräte und höheren Preisen anderer VR-Headsets entscheiden. In Deutschland führte diese Entwicklung zu einer Vertriebspause der Quest 2, da das Bundeskartellamt ein Missbrauchsverfahren gegen Facebook eingeleitet hat. Im vorliegenden Beitrag wird auf Basis einer Conjoint-Analyse untersucht, wie deutsche Endverbraucher dieses Dilemma wahrnehmen. Hierzu werden die relativen Wichtigkeiten von Datenschutzbestimmungen, Hardwareeigenschaften und Preisen für Kaufentscheidungen miteinander verglichen. Es ergeben sich drei verschiedene Marktsegmente mit unterschiedlichen Kaufentscheidungsheuristiken. Aus diesen Erkenntnissen resultieren sieben Handlungsempfehlungen, die VR-Herstellern, -Entwicklern, -Nutzern und Verbraucherschützern bei der verantwortungsvollen und weitreichenden Diffusion der VR-Technologie im Endverbrauchermarkt helfen sollen.

## Abstract (en)

Due to significant technological advances, virtual reality (VR) has increasingly established itself on the consumer market in recent years. In particular, the standalone headset Quest 2, manufactured by Facebook subsidiary Oculus, achieved high sales figures and became the top-selling VR glasses to date. At the same time, the privacy policy of Oculus, which links the use of the Quest 2 to a Facebook account, triggered a controversial debate among privacy experts. The introduction of this privacy policy faced consumers with a dilemma: They have to decide whether to share sensitive data with Facebook when using low-cost Oculus devices or pay higher prices for alternative VR headsets. The Federal Cartel Office then initiated data misuse proceedings against Facebook, resulting in a halt of sales of the Quest 2 in Germany. In this paper, we examine how German consumers perceive this dilemma. Based on a conjoint analysis, we compare the relative importance of privacy policies, hardware features and prices in the purchase decision for VR headsets. We identify three distinct market segments with different purchasing decision heuristics. The findings result in seven recommendations for action to help VR manufacturers, developers, and consumer advocates adopt VR technology responsibly and broadly in the consumer market.

## Das Oculus-Dilemma

Im vergangenen Jahrzehnt sorgten die erheblichen technologischen Fortschritte in den Bereichen Sensorik und Wearable Computing für zunehmende Reife und sinkende Preise von Virtual-Reality-Headsets (VR-Headsets). Führende Technologieunternehmen wie Facebook (nun: Meta) oder Google investierten seither massiv in das Hardware-Ökosystem und schufen damit die Grundlage für ein rapides Marktwachstum (Wohlgenannt et al. 2020). Insbesondere das Jahr 2020 war ein erfolgreicher Zeitraum für die VR-Industrie, da Konsumenten während der Kontaktbeschränkungen im Rahmen der Covid-19-Pandemie danach strebten, ihre Unterhaltungsbedürfnisse durch den Konsum virtueller Entertainment-Angebote zu befriedigen (Siani und Marley 2021). In diesem Zeitraum verzeichnete vor allem Facebooks Tochterunternehmen Oculus mit dem Standalone-Headset Quest 2 hohe Absatzzahlen, die auf disruptive Funktionalitäten (z. B. Hand-Tracking) sowie konsumentenfreundliche Preise zurückzuführen sind. Während diese Entwicklungen für VR-Enthusiasten erfreulich sind, zeigen sich Verbraucherschützer mit Blick auf Oculus neue Datenschutzbestimmung jedoch besorgt (Mir und Rodriguez 2020). Da die Nutzung von Oculus-Geräten seit Oktober 2020 an ein Facebook-Konto gekoppelt ist, hat Facebook Zugriff auf sensible Informationen wie Bewegungsdaten, die Rückschlüsse auf Verhaltensmuster, Emotionen und Identitäten der Benutzer zulassen (Miller et al. 2020, Oculus 2021).

Endverbraucher stehen seither vor einem Dilemma: Sie müssen sich zwischen der Preisgabe sensibler Daten an Facebook im Falle der Nutzung kostengünstiger Oculus-Geräte und höheren Preisen anderer VR-Headsets entscheiden. In Deutschland führte diese Entwicklung zu einem temporären Verkaufsstopp der Quest 2, da Facebook Teil eines Streitverfahrens mit dem Bundeskartellamt ist. Das Bundeskartellamt (2020) wirft Facebook vor, seine marktbeherrschende Position auszunutzen, um Oculus-Benutzer an seine Dienste zu binden. Diese Bindung verstoße gegen das Kopplungsverbot der Datenschutzgrundverordnung (DSGVO). Trotz dieser Kontroverse konnte Oculus zwischen Oktober 2020 und März 2021 nach Expertenschätzungen weltweit mehr als fünf Millionen Einheiten der Quest 2 absetzen. Die Absatzzahlen des Produktes übertreffen damit die kumulierten Verkäufe aller zuvor erschienenen Oculus-Geräte. Folglich avancierte die Quest 2 zum bisher meistverkauften VR-Headset (Lang 2021). Doch wie beurteilen deutsche Konsumenten diese Entwicklungen?

Empirische Endverbraucherstudien zeigen, dass hedonistische und pragmatische Benefits als Kernmotivation für die individuelle Adoption von VR fungieren, während Risiken wie etwa die Angst vor Datenmissbrauch die Diffusion bremsen (Herz und Rauschnabel 2019). Ähnlich dazu verdeutlicht der AR/VR Survey Report von Perkins Coie (2020), dass die Benutzerprivatsphäre die größte rechtliche Herausforderung für VR-Anbieter darstellt. Viele Hersteller versuchen sich zudem durch die Entwicklung und Patentierung innovativer Features von der Konkurrenz abzugrenzen. Diese Abgrenzung ist jedoch mit erheblichen finanziellen Risiken verbunden. Vor diesem Hintergrund ist es für VR-Anbieter essentiell, die Endverbraucherpräferenzen für VR-Headsets zu verstehen, um Kundensegmente zielgerichtet adressieren zu können. Daher lautet die diesem Beitrag zugrundeliegende Forschungsfrage:

Inwiefern beeinflussen Datenschutzbestimmungen im Vergleich zu Preisen und Hardwareeigenschaften die Kaufentscheidungen für Virtual-Reality-Headsets?

Zur Beantwortung dieser Forschungsfrage untersucht der vorliegende Beitrag die Endverbraucherpräferenzen für VR-Headsets auf Basis einer Conjoint-Analyse. Am Fallbeispiel Oculus wird analysiert, welchen Einfluss verschiedene Datenschutzbestimmungen im Vergleich zu Hardwareeigenschaften (z. B. Displayauflösung) und Preisen auf Kaufentscheidungen haben. Es resultieren drei Marktsegmente mit unterschiedlichen Präferenzmustern. Aus diesen Ergebnissen werden schließlich sieben Handlungsempfehlungen für VR-Anbieter, -Entwickler und -Benutzer sowie Verbraucherschützer abgeleitet, die zur weitreichenden und nachhaltigen Diffusion der Technologie dienen sollen.

## Virtual Reality im Endverbrauchermarkt

### Marktentwicklung und Hardwareeigenschaften

Im Jahr 2016 kam mit der Oculus Rift erstmals ein erschwingliches und performantes VR-Headset auf den Endverbrauchermarkt. Seither erfreut sich die immersive Technologie insbesondere im amerikanischen, chinesischen und europäischen Raum zunehmender Beliebtheit (Bezegová et al. 2018). Einer Bitkom-Befragung zufolge stieg die Nutzungsbereitschaft in der deutschen Bevölkerung zwischen den Jahren 2018 und 2020 von 17 auf 37 %. Zu den populärsten Einsatzfeldern gehören Computerspiele, virtuelle Reisen sowie der Multimediakonsum (Klöß 2020). Aufgrund der zunehmenden Popularität haben sich die globalen Absatzzahlen von VR-Headsets zwischen den Jahren 2016 und 2019 mit einer Zunahme von 18 Mio. auf 63 Mio. Einheiten mehr als verdreifacht (Mordor Intelligence 2021). Unter Einbezug der Nutzungsanforderungen lassen sich VR-Headsets in drei verschiedene Klassen unterteilen:

1. Mobile VR-Headsets beziehen sich auf kabellose Smartphone-Halterungen, die am Kopf befestigt werden können.
2. Standalone-VR-Headsets verfügen über eine eigene Recheneinheit, Sensoren und Grafikkarte und können autonom betrieben werden.
3. Stationäre VR-Headsets erfordern den Anschluss an einen externen Computer oder eine Konsole und sind zumeist auf High-End-Erlebnisse zugeschnitten.

Dieser Beitrag konzentriert sich auf autonome und stationäre VR-Headsets, da diese Geräte im Fokus der aktuellen Entwicklungen liegen. Tab. 1 gibt einen Überblick über acht VR-Headsets, die im Jahr 2021 angeboten werden.

**Tab. 1:** Übersicht aktueller VR-Headsets (Stand: Juli 2021)^a^

Name	Typ	Displayauflösung	Framerate [Hz]	Sichtfeld [°]	Gewicht [g]	Interaktion	Preis [€]
HP Reverb G2	Stationär	2160 × 2160	90	98	499	Controller	599
HTC Vive Focus 3	Standalone	2448 × 2448	90	120	N/A	Controller	1404
HTC Vive Pro 2	Stationär	2448 × 2448	90/120	120	850	Controller	799
Oculus Quest 2	Standalone	1832 × 1920	90	89	503	Controller mit Finger-Tracking; Hand-Tracking	399
Pico Neo 3	Standalone	1832 × 1920	90	98	350	Controller mit Finger-Tracking	550
Pimax 8K	Stationär	3840 × 2160	75/90	200	500	Controller	1499
Playstation VR	Stationär	1920 × 1080	120	90	610	Controller	299
Valve Index	Stationär	1440 × 1600	80/90/120	130	809	Controller mit Finger-Tracking	799

^a^Einige Hersteller behalten sich vor, die in Tab. 1 dargestellten Eigenschaften durch Software-Updates zu erweitern. Der Hersteller Pico hat beispielsweise für das dritte Quartal des Jahres 2021 ein Hand-Tracking-Update angekündigt

Für die Immersion sind insbesondere visuelle Faktoren wie die Auflösung, die Bildwiederholungsrate und das Sichtfeld von Bedeutung (Anthes et al. 2016). Hochwertige VR-Headsets (z. B. Pixmax 8K) verfügen bereits über eine 4K-Auflösung je Auge. Die Bildwiederholungsrate (engl. Framerate) liegt bei neueren Modellen über 90 Hz. Eine geringe Bildwiederholungsrate kann für den Benutzer zur Wahrnehmung von Ruckeln führen und wirkt sich negativ auf die Immersion aus. Das Sichtfeld gibt den Winkel an, den Nutzer durch das VR-Headset wahrnehmen. Da das natürliche Sichtfeld des Menschen 214 Grad beträgt, müssen Nutzer bei VR-Headsets, die maximal über ein Sichtfeld von 150 Grad verfügen, einen Verlust des peripheren Sehens in Kauf nehmen (Strasburger 2020). Ferner unterstützen VR-Headsets verschiedene Interaktionsmodalitäten. Die Interaktion erfolgt typischerweise über Motion Controller. Jüngere Entwicklungen in den Bereichen Finger- und Hand-Tracking ermöglichen es zudem, Finger- und Handbewegungen der Benutzer in die virtuelle Welt zu übertragen. Diese Interaktionsformen erhöhen den Realismus von Greif- und Wurfbewegungen. Für ein angenehmes Nutzererlebnis wird außerdem dem Gewicht der Endgeräte eine hohe Bedeutung beigemessen. Die in Tab. 1 aufgezeigten Standalone-Modelle wiegen beispielsweise maximal circa 500 g.

Die Anschaffungskosten von VR-Headsets variieren stark, angefangen bei kostengünstigen Geräten für Endverbraucher (z. B. Playstation VR, 299 €; Oculus Quest, 399 €) bis hin zu hochpreisigen B2B-Geräten (z. B. Pixmax 8K, 1499 €). Insbesondere Geräte mit einer hohen Displayauflösung sind in höheren Preisklassen zu verorten (z. B. Pixmax 8K). Zudem ist die Quest 2 mit einem Preis von 399 € trotz der Unterstützung von Finger- und Hand-Tracking vergleichsweise günstig. Diese Preisgestaltung ist darauf zurückzuführen, dass Mutterkonzern Facebook die Endgeräte durch App-Verkäufe und personalisierte Werbeanzeigen querfinanziert. Für die zielgruppenorientierte Werbung können die durch VR-Headsets erfassten Daten verwendet werden (Egliston und Carter 2020). Folglich zahlen Endverbraucher Oculus-Geräte nicht nur monetär, sondern auch durch die Preisgabe ihrer Daten. Um ein tiefergehendes Verständnis für dieses Geschäftsmodell und die damit einhergehenden Datenschutzaspekte zu gewinnen, werden diese nachfolgend beleuchtet.

### Datenschutzbezogene Besonderheiten

Zur Darbietung einer immersiven Erfahrung erfassen VR-Headsets eine Vielzahl von Informationen über spezifische Sensoren. Stationäre Systeme (z. B. HTC Vive) verwenden Accelerometer, Laser-Positionsmesser und Gyrosensoren, um die Positionen und Bewegungen der Benutzer zu erfassen. Im Gegensatz dazu nutzen Standalone-Systeme vorrangig Inside-Out-Tracking, das die Bewegungen mittels integrierter Kameras erfasst. Die Quest 2 verfügt beispielsweise über vier Ultra-Weitwinkelkameras, die das Umfeld sowie die Bewegungen des Benutzers erkennen und Hand-Tracking ermöglichen. Diese Sensordaten werden jedoch nicht nur lokal auf den Geräten verarbeitet. Einer Netzwerkverkehrsanalyse zufolge werden die Trackingdaten auch für analytische Zwecke an Facebook-Server gesendet (Trimananda et al. 2021). Langfristig soll Facebook diese Informationen nach Experteneinschätzungen für das Training von KI-Modellen einsetzen, um Nutzerverhalten präziser vorhersagen zu können (Bastian 2020).

Neben diesen VR-spezifischen Daten erfassen Oculus-Endgeräte auch persönliche Informationen. Um auf die Oculus-VR-Services zugreifen und Apps über den Oculus Store beziehen zu können, müssen sich Benutzer mit einem persönlichen Account auf den Geräten einloggen. Während hierfür bis September 2020 ein von Facebook losgelöster Oculus-Account vorgesehen war, benötigen Neukunden seit Oktober 2020 einen aktiven Facebook-Account. Seither behält sich Facebook in den Datenschutzbestimmungen für Oculus-Geräte vor, die in Tab. 2 aufgeführten Daten zu verarbeiten.

**Tab. 2:** Erfasste Informationen. (Quelle: Oculus 2021)

Typ	Kurzbeschreibung	Beispiel(e)
Physische Funktionen	Charakteristika und Körpermaße	*Geschätzte Handgröße*
Inhalte	Aufnahmen und Werke der Benutzer inkl. Meta-Daten	*Avatare, 3D-Objekte, Datum und Uhrzeit*
Cookies	Website-Einstellungen	*Sprache, E‑Mailadressen, Namen*
Interaktionen	Ausgeführte Funktionen	*Dauer der Browser-Nutzung*
Umgebungs‑, Dimensions- und Bewegungsdaten	Informationen über die Umgebung und Bewegung des Benutzers	*Räumliches Modell des Spielbereichs, Handbewegungen*
Informationen von Drittparteien	Von Entwicklern, App-Anbietern und Marketingpartnern bereitgestellte Daten	*Verstöße gegen Content-Richtlinien, die von Dritten gemeldet wurden*
Technische Systemdaten	Daten zur Nutzung von Oculus-Produkten	*IP-Adresse, Nutzer-ID, Absturzprotokolle, lokale Dateipfade*

Facebook wertet Teile dieser Daten nach eigenen Angaben zur Verbesserung und Personalisierung der eigenen Produkte und Services aus. Das Facebook-Geschäftsmodell ist darauf ausgerichtet, durch den Verkauf zielgruppenorientierter Werbeanzeigen Umsätze zu erzielen (Funk et al. 2012). Auch Oculus platziert seit dem Jahr 2021 immersive Werbeanzeigen in Apps und Spielen. In der aktualisierten Datenschutzbestimmung behält sich Facebook außerdem vor, Teile dieser Daten an weitere Unternehmen der Facebook-Gruppe zu übermitteln (Oculus 2021).

Laut Bundeskartellamt (2021) setzt sich Facebook mit dieser Datenschutzbestimmung auf zwei Ebenen über den regulatorischen Rahmen hinweg. Erstens verstößt der Social-Media-Konzern nach Ansicht des Bundeskartellamtes mit dem Facebook-Zwang gemäß Art. 7 Abs. 3 gegen das Kopplungsverbot der DSGVO, da er seine marktbeherrschende Position ausnutze, um Oculus-Nutzer an seine Dienste zu binden. Zweitens führe Facebook unerlaubt Nutzerdaten aus verschiedenen Quellen (z. B. Oculus VR und Facebook) zusammen, ohne die aktive Zustimmung der Nutzer einzuholen. Das Bundesskartellamt hatte die Erstellung von Super-Profilen aufseiten von Facebook bereits im Jahr 2020 kritisiert. Zu diesem Zeitpunkt bezog es sich auf die Zusammenführung von Nutzerdaten aus Instagram‑, Whatsapp- und Facebook-Konten. Parallel zur Einleitung des Missbrauchsverfahrens im Jahr 2021 stellte Oculus den Verkauf der Quest 2 in Deutschland proaktiv temporär ein. Ein Import des Produktes aus dem EU-Ausland war in diesem Zeitraum über Vertriebsplattformen wie z. B. amazon.it dennoch möglich. Mit einem gültigen Facebook-Account konnten deutsche Benutzer derweil alle Funktionen der Quest 2 abrufen. Unklar bleibt bislang jedoch, inwiefern diese durchaus umstrittene Datenschutzbestimmung die Kaufbereitschaft im Vergleich zu Hardwareeigenschaften und Preisen beeinflusst.

## Präferenzmessung mittels Conjoint-Analyse

Um die zuvor skizzierten Forschungslücke zu schließen, wurde für den vorliegenden Beitrag eine auswahlbasierte Conjoint-Analyse durchgeführt. Diese Präferenzmessmethode stammt ursprünglich aus der Marketingforschung und basiert auf der Annahme, dass Konsumenten ein Produkt als eine Kombination von Attributen (z. B. Preis) sehen, die verschiedene Ausprägungen haben (z. B. €10, €20; Green und Srinivasan 1978). Im Rahmen einer auswahlbasierten Conjoint-Analyse durchlaufen die Befragten verschiedene Auswahlsituationen, in denen sie sich zwischen fiktiven Produktangeboten (Stimuli) entscheiden. Auf Basis der getroffenen Entscheidungen werden anschließend die Teilnutzenwerte einzelner Ausprägungen sowie die relative Wichtigkeiten der Attribute berechnet (Backhaus et al. 2016).

Der vorliegenden Untersuchung liegt ein vierstufiges Vorgehen zugrunde. Im ersten Schritt wurde eine Vorstudie durchgeführt, um die Attribute und Ausprägungen der Stimuli zu bestimmen. Im zweiten Schritt wurde ein Online-Fragebogen konzipiert und implementiert. Anschließend wurden eine Hierarchical-Bayes-Schätzung (HB-Schätzung) und eine Clusteranalyse durchgeführt, um die Teilnutzenwerte sowie die Marktsegmente zu bestimmen.

### Auswahl von Attributen und Ausprägungen

Für die Auswahl der Attribute und Ausprägungen sichteten die Autoren zunächst Online-Shops sowie VR-Blogs. Die Vorauswahl der Attribute wurden anschließend in acht Interviews mit VR-Benutzern validiert und priorisiert. Die Befragten waren zum Interviewzeitpunkt durchschnittlich 24,6 Jahre alt. Von den acht Befragten waren zwei weiblich und sechs männlich.

Während der Interviews betonten alle Befragten die Relevanz des Preises für die Kaufentscheidung. Sie gaben an, dass sie im Durschnitt maximal 900 € für ein VR-Headset bezahlen würden. Unter Einbezug der in Abschn. 2 verglichenen VR-Headsets wurden die Ausprägungen 400 €, 650 € und 900 € in das Versuchsdesign integriert. Neben dem Preis sind für fünf Interviewpartner die Interaktionsmodalitäten von besonderer Bedeutung. Insbesondere Hand-Tracking wurde von den Befragten als wichtige Ausprägung genannt. Außerdem betonten sechs Befragte die Wichtigkeit der Displayqualität. Der Full-HD-Standard wurde in den Interviews als Mindestanforderung für ein immersives Erlebnis hervorgehoben. Ferner berichteten drei Befragte aufgrund des Facebook-Zwangs, der seit Oktober 2020 für die Nutzung von Oculus-Geräten implementiert wurde, von Datenschutzbedenken. Zwei Befragte gaben an, dass sie bereit wären, eine zusätzliche Gebühr zu zahlen, um die Privatsphäre-Einstellungen anpassen zu dürfen. Vor diesem Hintergrund wurde die Ausprägung Individualisierbar integriert und mit den zuvor von Oculus angebotenen Datenschutzbestimmungen komplementiert. Schließlich betonten sechs Befragte die Relevanz der Nutzungsanforderungen. Aufgrund der niedrigen Performanz mobiler VR-Headsets wird ausschließlich zwischen stationären und Standalone-Systemen unterschieden. Tab. 3 gibt einen Überblick über die Attribute und Ausprägungen.

**Tab. 3:** Attribute und Ausprägungen

Attribut	Ausprägungen (Beschreibung)
Preis	**1) 400** **€**
	**2) 650** **€**
	**3) 900** **€**
Interaktion	**1) Controller: **Benutzer können mit Controllern, einschließlich Tasten, Analogsticks und Touchpads, in der virtuellen Realität interagieren
	**2) Controller mit Finger-Tracking: **Benutzer können mit Controllern, die neben Tasten auch Fingergestenerkennung unterstützen, mit der virtuellen Realität interagieren
	**3) Hand-tracking: **Neben Controllern, die Finger-Tracking unterstützen, können Benutzer über Handgesten mit der virtuellen Umgebung interagieren
Displayqualität	**1) Niedrig. **Die Displayauflösung entspricht dem Full-HD-Standard
	**2) Moderat. **Die Auflösung liegt zwischen den Standards Full HD und 4K
	**3) Hoch. **Die Display-Auflösung entspricht dem 4K-Standard
Datenschutzbestimmung	**1) Facebook-Login: **Benutzer benötigen ein Facebook-Konto, um das Gerät zu verwenden. Oculus kann Ihre VR-Aktivitäten und Informationen mit Facebook teilen, um personalisierte Dienste (z. B. Werbung) anzubieten
	**2) Oculus-Login: **Benutzer benötigen ein Oculus-Konto, um das Gerät zu verwenden. Lediglich Oculus kann ihre Aktivitäten und Informationen nutzen, um seine Produkte zu verbessern und personalisierte Dienste (z. B. Werbung) anzubieten
	**3) Individualisierbar:** Benutzer können selbst entscheiden, wie sie sich anmelden und welche Daten sie bei der Nutzung der Dienste mit dem Hersteller und dessen Mutterkonzern Facebook teilen
Nutzungsanforderung	**1) Standalone-System: **Das Gerät verfügt über eine eigene Recheneinheit und kann ohne Zusatzgeräte (z. B. PC) verwendet werden
	**2) Stationäres System:** Zur Nutzung muss das Gerät mit einem PC verbunden werden, der die Rechenleistung zur Verfügung stellt

Diese Attribute und Ausprägungen bildeten die Grundlage für die randomisierte Erzeugung der Auswahlsituationen. Mit der Statistiksoftware R Studio und der Erweiterung Algorithmic Experimental Design wurden insgesamt 17 Auswahlsituationen generiert. Jede Auswahlsituation enthält zwei verschiedene Stimuli sowie eine No-Choice-Option, die aussagt, dass sich die Befragten zwischen den zwei Produktalternativen nicht entscheiden können.

### Befragung und Marktsegmentierung

Die 17 Auswahlsituationen wurden in einen vierteiligen Online-Fragebogen eingebettet. Im ersten Teil wurden die Teilnehmer gebeten, sich vorzustellen, dass sie eine VR-Brille kaufen wollen und ein Freund bereits Systeme der Marke Oculus vorausgewählt hat. Der zweite Teil beinhaltete die 17 Auswahlaufgaben. Im dritten Teil wurden die Befragten darum gebeten, eine Priorisierung der Attribute vorzunehmen und die Rangfolge zu begründen. Abschließend wurden soziodemografische Merkmale erhoben.

An der Umfrage nahmen im Frühjahr 2021 insgesamt 255 Wirtschaftsinformatik- und Wirtschaftswissenschaftenstudierende einer deutschen Universität teil. Im Screening-Prozess wurden 30 unvollständige Fragebögen exkludiert. Von den 225 Befragten waren 59,31 % männlich und 40,69 % weiblich. Im Durchschnitt waren die Teilnehmenden zum Zeitpunkt der Befragung 20,94 Jahre alt und etwa jeder zweite Befragte (47,56 %) hatte bereits Erfahrung mit VR-Headsets.

Die anschließende Datenauswertung erfolgte mithilfe der Statistiksoftware R und dem Paket *bayesm*, das HB-Schätzungen unterstützt. Im zweiten Schritt führten die Autoren auf Basis der individuell geschätzten Teilnutzenwerte eine zweistufige Clusteranalyse in SPSS durch, um verschiedene Marktsegmente mit homogenen Präferenzen zu identifizieren.

## Untersuchungsergebnisse

### Segmentspezifische Analyse

Durch die a‑posteriori-Segmentierung wurden drei verschiedene Marktsegmente ermittelt.^1^ Das größte Segment bildet dabei Segment 1, zu dem 40,89 % der Stichprobe gehören. Weitere 22,22 % der Stichprobe sind in Segment 2 zu verorten, während die verbleibenden 36,89 % der Stichprobe Mitglied von Segment 3 sind.

**Abb. 1:**
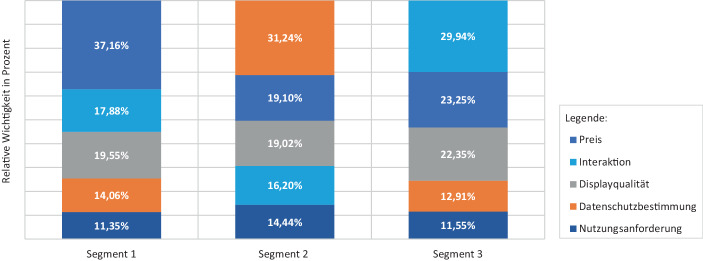
Relative Wichtigkeiten der Produktattribute je Marktsegment

Im Durchschnitt bevorzugen alle drei Segmente VR-Headsets für einen Preis von 400 €, die Hand-Tracking unterstützen und über eine hohe Displayqualität verfügen. Außerdem präferieren die drei Segmente die individualisierbare Datenschutzbestimmung und Standalone-Systeme. Wie in Abb. 1 dargestellt, unterscheiden sich die Segmente hinsichtlich der durchschnittlichen relativen Wichtigkeiten^2^ der Produktattribute jedoch signifikant voneinander.

#### Preisjäger:

Segment 1 (40,89 % der Befragten) strebt in seinen Auswahlentscheidungen nach preisgünstigen VR-Headsets. Folglich misst es dem Attribut *Preis* mit einer relativen Wichtigkeit von 37,16 % den größten Stellenwert zu. Zudem sind für Segment 1 die *Displayqualität* (19,55 %) sowie die *Interaktion* (17,88 %) die zweit- und drittwichtigsten Attribute. Die relativen Wichtigkeiten dieser Attribute sind etwa halb so groß wie die des Preises. Dabei ist Segment 1 durch eine hohe Aversion gegen niedrige Displayauflösungen gekennzeichnet, was durch den kleinsten Teilnutzenwert dieser Ausprägung über alle Cluster hinaus indiziert wird (vgl. Tab. 4 im Anhang). Aufgrund des Fokus auf preisgünstige Geräte mit hochauflösenden Displays und Hand-Tracking-Kapazitäten beeinflussen die *Datenschutzbestimmungen* die Kaufentscheidungen mit einer relativen Wichtigkeit von 14,06 % in Segment 1 deutlich schwächer als in Segment 2. Angesichts dieser untergeordneten Bedeutung der Datenschutzbestimmung ist Segment 1 vermutlich dazu bereit, dem Facebook-Zwang einzuwilligen, da Oculus günstigere Endgeräte als seine Konkurrenten anbietet. Gleichzeitig könnte der durch den Facebook-Zwang induzierte Nutzenverlust durch das Angebot einer hohen Displayqualität oder durch die Unterstützung von Hand-Tracking kompensiert werden. Überraschenderweise hat die *Nutzungsanforderung* in allen drei Segmenten nur einen untergeordneten Einfluss auf die Kaufentscheidungen. Die relative Wichtigkeit dieses Attributes beträgt in Segment 1 11,35 %.

#### Datenschützer:

Segment 2 (22,22 % der Befragten) priorisiert in seinen Kaufentscheidungen den Schutz seiner Daten: Der Einfluss der Datenschutzbestimmungen ist mit einer relativen Wichtigkeit von 31,24 % in diesem Segment mehr als doppelt so hoch wie in den Segmenten 1 und 2. Damit hat das Attribut *Datenschutzbestimmung* für dieses Segment in der vorliegenden Studie die höchste Relevanz für die Kaufentscheidung. Die Teilnutzenwerte der einzelnen Datenschutzbestimmungen untermauern, dass Segment 2 sehr sensibel auf den Facebook-Zwang reagiert. Die Datenschutzbestimmung, die an ein Facebook-Konto gekoppelt ist, stiftet als einzige Ausprägung im zugehörigen Attribut einen negativen Teilnutzenwert. Somit strebt Segment 2 während der Kaufentscheidungen danach, die Hoheit über seine Daten zu behalten, indem es die Weitergabe von Informationen an Oculus oder Facebook vermeidet. Aufgrund dieser Heuristik besitzt das Attribut *Preis* in Segment 2 mit einer relativen Wichtigkeit von 19,10 % eine deutlich geringere Relevanz als in Segment 1. Folglich sind Mitglieder von Segment 2 dazu bereit, einen höheren Preis für VR-Headsets mit einem hohen Datenschutz zu bezahlen. Gleichzeitig zeigen die technischen Attribute für Segment 2 weniger Relevanz als in den anderen beiden Segmenten. So fließen die *Displayqualität* sowie die *Interaktion* jeweils mit einer relativen Wichtigkeit von 19,02 bzw. 16,20 % in die Kaufentscheidung ein und bilden damit die dritt- bzw. viertwichtigsten Attribute.

#### Tech-Enthusiasten:

Segment 3 (36,89 % der Befragten) legt in seinen Auswahlentscheidungen einen hohen Wert auf technische Eigenschaften und gewichtet die zugehörigen Attribute am stärksten. Dabei hat das Attribut *Interaktion* mit einer relativen Wichtigkeit von 29,94 % den größten Einfluss auf die Kaufentscheidung. Insbesondere die Unterstützung von Hand-Tracking ist für dieses Segment wichtig. Gleichzeitig beeinflusst das Attribut *Displayqualität* die Auswahlentscheidungen mit einer relativen Wichtigkeit von 22,35 % stärker als in Segmenten 1 und 2. Insgesamt besitzen diese beiden Produkteigenschaften damit eine relative Wichtigkeit von mehr als 52 %, während das Attribut *Preis* deutlich geringere Relevanz aufweist als in den anderen beiden Segmenten. Aufgrund der Präferenz für Systeme mit moderner technischer Ausstattung ist die Relevanz des Attributes *Datenschutzbestimmung* in Segment 3 clusterübergreifend am geringsten (12,91 %).

### Motive zur Priorisierung und Vernachlässigung der Attribute

**Abb. 2:**
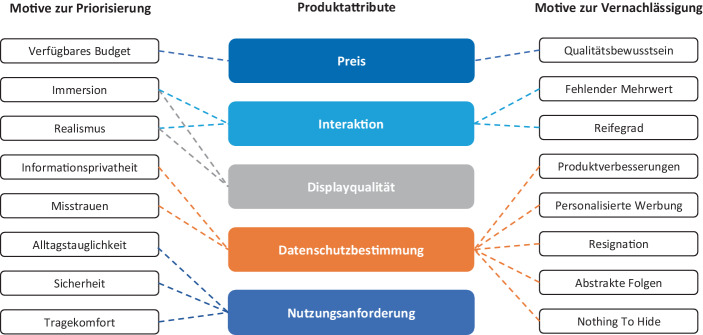
Motive zur Priorisierung und Vernachlässigung der Produktattribute

Um die verschiedenen Motivationen zur Priorisierung und Vernachlässigung der Attribute zu verstehen, wurden die Freitextantworten ausgewertet. In diesem Prozess wurden 16 verschiedene Einflussfaktoren identifiziert. Abb. 2 visualisiert die Einflussfaktoren und ihre Wirkungen auf die Gewichtung der Attribute.

#### Preis:

Die hohe Relevanz des Preises in Segment 1 ist primär auf das verfügbare Budget der Befragten zurückzuführen. So wurden in den Freitextfeldern wiederkehrend Preisgrenzen zwischen 500 und 600 € genannt. Komplementär dazu betonten insbesondere Mitglieder von Segment 3 ihr Qualitätsbewusstsein. Sie seien bereit dazu, höhere Preise für „hochqualitative, innovative und komplexe Produkte“ zu bezahlen, um „langfristig etwas von diesen Produkten zu haben“. Schließlich gehe mit einer Preissteigerung eine höhere Qualität sowie eine gesteigerte Langlebigkeit technischer Produkte einher.

#### Interaktion:

Hinsichtlich der hohen Bedeutung der Interaktionsmodalitäten betonten insbesondere Befragte aus Segment 3 die Rolle der Immersion, um „tiefer in die virtuelle Welt eintauchen und das Erlebnis mehr genießen zu können“. Dieses „deutlich bessere Erlebnis“ sei ein entscheidender Vorteil der VR-Technologie gegenüber konventionellen Benutzerschnittstellen. Ebenso verhelfe Hand-Tracking dazu, die „virtuelle Welt realistischer wahrzunehmen“. Für Befragte, die der Interaktion eine untergeordnete Rolle zuweisen, sei der Mehrwert von Finger- und Hand-Tracking nicht erkennbar. Zudem zweifelten Teilnehmer der Studie an, ob neue Interaktionsformen, wie beispielsweise Hand-Tracking, aus technischer Sicht ausgereift genug sind, um eine „zuverlässige und präzise Interaktion mit der virtuellen Umgebung“ zu unterstützen.

#### Displayqualität:

Neben der Interaktion wird der Displayqualität in allen Segmenten zur Sicherstellung der Immersion und des Realismus eine hohe Bedeutung beigemessen. So gab ein Befragter beispielsweise an, dass eine „verpixelte Darstellung der virtuellen Umgebung zu einer unrealistischen Darstellung beiträgt“. Grundsätzlich fiel bei der Durchsicht der Kommentare clusterübergreifend ein homogenes Meinungsbild zur Relevanz der Displayqualität auf.

#### Datenschutzbestimmung:

Als Motive zur Priorisierung der Datenschutzbestimmungen in Segment 2 gelten insbesondere Misstrauen gegenüber dem Technologiekonzern Facebook sowie grundsätzliche Bedenken hinsichtlich der Aufgabe der Informationsprivatheit. Statements wie „Ich möchte nicht, dass Facebook meine Daten besitzt, nur, weil ich seine Produkte nutze.“ verdeutlichen die kritische Haltung der Datenschützer, die den Facebook-Zwang mitunter als „unheimlich“ und „besorgniserregend“ bezeichnen. Komplementär dazu sehen beispielsweise Mitglieder von Segment 3 in der Datenverarbeitung durch Facebook Vorteile wie „personalisierte Werbung“ und „verbesserte Algorithmen“. Ferner fielen in den Freitextfeldern Anzeichen für die Mechanismen Resignation, Abstraktheit der Folgen und Nothing to Hide auf, die bereits von den Autoren Rauschnabel et al. (2018) als Motive zur Aufgabe der eigenen Privatsphäre bei der privaten Nutzung von Augmented Reality Smart Glasses identifiziert wurden. Da ein großer Teil der Befragten beispielsweise bereits Mitglied bei „Facebook, Instagram, Amazon, etc.“ ist, sei „der Datenschutz schon vor der Nutzung nicht mehr vorhanden und relevant“. Somit falle „die Überwachung über VR-Headsets nicht mehr ins Gewicht“. Andere Teilnehmer der Studie hätten nach eigener Aussage schlichtweg „nichts zu verbergen“.

#### Nutzungsanforderung:

Im Kontext der Nutzungsanforderungen betonten die Befragten abschließend die Vorteile von Standalone-Headsets. Insbesondere Usability-Vorteile (z. B. höhere Flexibilität, mehr Bewegungsfreiräume durch fehlende Kabel und ortsunabhängige Einsatzmöglichkeiten) sprächen aus Sicht der Befragten für eine Wahl von Standalone-Headsets. Gleichzeitig trage der Verzicht auf „lästige Kabel“ zu einer erhöhten Sicherheit während der Nutzung bei und stelle darüber hinaus den Tragekomfort sicher.

## Handlungsempfehlungen für Akteure aus dem Virtual-Reality-Ökosystem

Zweifellos birgt die VR-Technologie aufgrund der Vielzahl integrierter Sensoren neue datenschutzbezogene Herausforderungen, denen sich sowohl Hardwareanbieter als auch Entwickler im Spannungsfeld zwischen einer möglichst immersiven User Experience, ihren Geschäftsmodellen und regulatorischen Rahmenbedingungen stellen müssen. Der vorliegende Beitrag zeigt, dass Endverbraucher diese Spannungsfelder sehr heterogen wahrnehmen. Die Kombination aus einer Conjoint-Befragung und einer Clusteranalyse führt zu drei verschiedenen Marktsegmenten mit unterschiedlichen Präferenzmustern. Um Akteure aus dem VR-Ökosystem, zu denen Soft- und Hardwareanbieter, Entwickler sowie Endverbraucher gehören (Schuir et al. 2020), bei einer weitreichenden und verantwortungsvollen Diffusion der VR-Technologie im Massenmarkt zu unterstützen, werden nachfolgend segmentspezifische Handlungsempfehlungen (HE) abgeleitet.

### HE1: Produktbereitstellung über Rental Services für preisbewusste Konsumenten

Für rund 40 % der Befragten – die Preisjäger – gilt der Verkaufspreis eines VR-Headsets als wichtigstes Kaufkriterium. Insbesondere Produkte mit einem Preis über 650 € zieht dieses Segment nicht in Betracht, da die zugehörigen Teilnutzenwerte den wahrgenommenen Gesamtnutzen senken. Tatsächlich übertrifft die Mehrheit der in Abschn. 2 verglichenen VR-Headsets jedoch diese Preisgrenze (z. B. Valve Index). Zur Senkung dieser finanziellen Einstiegsbarrieren bietet es sich an, VR-Headsets über Rental Services zur Verfügung zu stellen. Bisher werden VR-Headsets insbesondere im B2B-Geschäft vermietet. Da VR-Endgeräte vorrangig online erwerbbar sind, können sich preisbewusste Konsumenten durch die Bereitstellung von VR-Headsets über Rental-Services zu vergünstigten Konditionen mit der Technologie vertraut machen und erste Hands-On-Erfahrungen sammeln. Die positiven Erfahrungen mit der VR-Technologie könnten bei diesen Konsumenten mittelfristig zu einer erhöhten Zahlungsbereitschaft für VR-Headsets beitragen.

### HE2: Wertschöpfung durch konsumentenfreundliche Datenschutzbestimmungen

Insgesamt besitzen Datenschutzbestimmungen für rund ein Viertel der Stichprobe – die Datenschützer – eine hohe Relevanz in den simulierten Kaufentscheidungen. Der Oculus-Datenschutzbestimmung, die ein Facebook-Konto mit Klarnamen vorsieht, wird dieses Segment aufgrund seiner Präferenz für VR-Headsets mit einer hohen Informationsprivatheit vermutlich nicht zustimmen. Eine Querfinanzierung von Oculus-Endgeräten durch personalisierte Werbeanzeigen lässt sich in diesem Marktsegment offenbar nicht durchsetzen. Vielmehr deuten die Ergebnisse darauf hin, dass Segment 2 aufgrund seiner geringen Preissensibilität dazu bereit ist, höhere Preise für eine hohe Informationsprivatheit zu bezahlen. Dieses Präferenzmuster wirft aus Anbieterperspektive zwei Wertschöpfungsmöglichkeiten auf. Erstens können sich Oculus-Konkurrenten durch die Hervorhebung einer DSGVO-konformen Informationsverarbeitung aktiv vom Marktführer abgrenzen. Zweitens können Software- und Hardwareanbieter, deren Geschäftsmodell auf die Erfassung und Analyse von Kundendaten ausgerichtet ist, alternative Einnahmequellen etablieren. So kann Oculus beispielsweise sein Nutzenversprechen (engl. Value Proposition) durch individualisierbare Datenschutzeinstellungen anreichern und diese gleichzeitig monetarisieren, wenn Konsumenten nicht damit einverstanden sind, ihre Daten zu Werbe- oder Produktverbesserungszwecken zu teilen. Oculus bietet beispielsweise bereits gegen einen Aufpreis von 400 US-Dollar eine Business-Variante der Quest 2 an, für die kein Facebook-Konto notwendig ist. Statt einer Einmalzahlung können zur Monetarisierung wie in sozialen Netzwerken (z. B. LinkedIn) oder Streaming-Diensten (z. B. Youtube) beispielsweise bezahlbare Premium-Accounts angeboten werden, für die monatliche Gebühren entfallen.

### HE3: Verbraucherschutz durch technologiespezifische Datenschutzgütesiegel

Hinsichtlich der relativen Wichtigkeit der Datenschutzbestimmung in Segment 2 ist anzumerken, dass die gesamte Stichprobe im ersten Teil der Umfrage ausführlich über die durch VR-Headsets erfassten Informationen sowie die Informationsflüsse zwischen Facebook und Oculus aufgeklärt wurde. In der Praxis erwerben Konsumenten jedoch zunächst ein VR-Headset und stimmen erst bei der initialen Nutzung gemäß des Prinzips der informierten Einwilligung den Datenschutzbestimmungen und Nutzungsbedingungen zu. Diese informierte Einwilligung setzt im Sinne des Bundesdatenschutzgesetzes eine transparente und leicht verständliche Erklärung der Datenerfassung-, -speicherung und -verarbeitung voraus. Die tatsächlich implementierten Datenschutzbestimmungen sind durchaus als komplexe Dokumente einzustufen, die nicht sorgfältig gelesen werden und Nutzer häufig mit Digital-Nudging-Techniken beeinflussen (Utz et al. 2019). Angesichts der Bandbreite sowie der Sensibilität der durch VR-Technologien erfassten (z. B. biometrischen) Daten, wird an dieser Stelle empfohlen, technologiespezifische Datenschutzgütesiegel zu entwickeln. Diese Datenschutzgütesiegel sollten zur transparenten Verbraucheraufklärung vor der Kaufentscheidung auf Hardwarevertriebsplattformen gelistet werden. Aufgrund der Signifikanz datenschutzrechtlicher Fragestellungen sind Tech-Blogs (z. B. MIXED) teilweise bereits dazu übergegangen, Datenschutzbestimmungen in ihren Kaufratgebern zu berücksichtigen. Angesichts der zunehmenden Reife und Verfügbarkeit von Sensortechnologien wird diese Form der Verbraucheraufklärung voraussichtlich auch zukünftig von steigender Relevanz sein.

### HE4: Datenschutz durch Präventionsmaßnahmen

VR-Benutzer mit einer ausgeprägten IT-Affinität, die nicht auf neue Oculus-Produkte verzichten wollen, können Präventionsmaßnahmen ergreifen, um die eigenen Daten zu schützen. Diese Maßnahmen werden in sozialen Medien (z. B. Twitter und Reddit) diskutiert. Ein Beispiel hierfür ist die Software Pi-Hole, die Datenverkehr für unerwünschte Tracking- oder Werbedienste unterbindet. Hierfür wendet die Software vordefinierte Filterregeln an und sendet bei Serveranfragen, die mit Tracking und Werbung assoziiert werden, leere Rückmeldungen. Dadurch sind beispielsweise Werbebanner für die Benutzer nicht mehr sichtbar. Ebenfalls können sich Oculus-Benutzer durch alternative Apps schützen. Der Firefox-VR-Browser integriert beispielsweise einen erweiterten Tracking-Schutz und schützt nach Herstellerangaben damit die Informationen seiner Benutzer besser.

### HE5: Entwicklung normativer Leitlinien für das VR-Ökosystem

Die VR-Technologie wirft unbestritten neue rechtliche und ethische Fragestellungen auf, die in Produktentwicklungsprozessen berücksichtigt werden müssen. Soft- und Hardwareentwicklern kommt vor diesem Hintergrund eine hohe gesellschaftliche Verantwortung zu. Da sich die Diffusion immersiver Produktangebote im Jahr 2021 noch im formativen Stadium befindet, gibt es bislang beispielsweise kaum Erfahrungen und Standards, die für die Entwicklung spezifischen Datenschutzbestimmungen herangezogen werden können (XR Safety Initiative 2020). Dies gilt nicht nur für den Datenschutz, sondern auch für die normative Gestaltung von Avataren (z. B. hinsichtlich der Diversität), Benutzererfahrungen (z. B. zur Reduktion von Simulationskrankheit) und Sicherheitsmechanismen (z. B. aufgrund der isolierenden Hardwarecharakteristika, Adams et al. 2019). An dieser Stelle bietet die interdisziplinäre Forschung vielversprechende Ressourcen zur Bewältigung ethischer, rechtlicher und sozialer Herausforderungen, die durch die Entwicklung normativer Leitlinien für das VR-Ökosystem ausgeschöpft werden können.

### HE6: Zusatzverkäufe durch innovative Interaktionsmodalitäten

Für etwa vier von zehn Befragten – die Tech-Enthusiasten – gelten die Attribute Interaktion und Displayqualität in der vorliegenden Studie als wichtigste Kaufkriterien eines VR-Headsets und fließen mit mehr als 50 % in die Auswahlentscheidungen ein, während Preise in diesem Segment eine unterdurchschnittliche Relevanz besitzen. Aus VR-Hardware-Anbietersicht deutet dieses segmentspezifische Ergebnis auf eine erhöhte Zahlungsbereitschaft für Endgeräte mit einem hohen Innovationsgrad (z. B. mit 8K-Displays, Eye-Tracking, Brain-Computer-Interfaces) hin. Durch das Angebot von Premium-Devices, die diese Anforderungen erfüllen, können sich Marktteilnehmer von Oculus abgrenzen und dieses Marktsegment zielgerichtet erschließen. Ebenso können gerätespezifische Erweiterungen, die neue Interaktionsformen ermöglichen, entwickelt und angeboten werden, um Zusatzverkäufe zu generieren. Beispielsweise bietet VIVE einen Gesichts-Tracker an, der die Modelle der VIVE Pro Serie nachträglich zur Erkennung von Mimik befähigt.

Analog dazu können sich Software-Entwickler durch die Unterstützung innovativer Interaktionsmodalitäten (z. B. Hand-Tracking) von Konkurrenzangeboten abgrenzen. Grundsätzlich unterstreichen die Teilnutzenwerte ein hohes Interesse an Hand-Tracking-Anwendungen. Da die Mehrheit der angebotenen Apps (z. B. im Oculus Store) bislang kein Hand-Tracking unterstützt, können durch die Berücksichtigung dieser Konsumentenpräferenzen zusätzliche App-Verkäufe generiert werden. Aus technischer Sicht bietet das Mixed Reality Toolkit beispielsweise ein Rahmenwerk zur Unterstützung von Hand-Tracking in Unity 3D (siehe Vogel et al. 2021). Da In-App-Käufe für mehr als die Hälfte aller VR-Softwareanbieter zur wichtigsten Einnahmequelle gehören (Perkins Coie 2020), kann die Gestensteuerung beispielsweise als Zusatzservice über In-App-Käufe monetarisiert werden.

### HE7: Konzeption segmentspezifischer Marketingstrategien

Abschließend unterstreichen die Ergebnisse der vorliegenden Untersuchung die Heterogenität von Konsumentenpräferenzen für VR-Headsets. Nachdem VR ab dem Jahr 2016 vorrangig unter Gamern Aufmerksamkeit erregte, nahm das Nutzungsinteresse in der breiten Bevölkerung insbesondere im Jahr 2020 zu. Um die Diffusion der virtuellen Realität im Massenmarkt zu fördern, bietet die vorliegende Untersuchung Anhaltspunkte zur Konzeption segmentspezifischer Marketingstrategien. Die analysierten Segmente können dabei helfen, die Präferenzmuster im Endverbrauchermarkt zu verstehen, während die identifizierten Motive Anhaltspunkte dafür geben, wie die Segmente anzusprechen sind. So sollten für Segment 3 beispielsweise Faktoren wie die Immersion und der Realismus in der Marketingkommunikation hervorgehoben werden, während Segment 1 vorrangig durch Niedrigpreisstrategien erreicht werden kann.

## Schlussbetrachtung und Ausblick

Der vorliegende Beitrag stellt die Ergebnisse einer Conjoint-Analyse mit 225 Befragten vor. Er präsentiert drei verschiedene Segmente aus dem VR-Markt, die sich in ihren Kaufentscheidungsheuristiken für VR-Headsets erheblich voneinander unterscheiden. Während für die Preisjäger (Segment 1) die Anschaffungskosten das wichtigste Kaufkriterium darstellen, sind die Datenschützer (Segment 2) dazu bereit, höhere Preise für VR-Headsets mit einem hohen Datenschutz zu bezahlen. Im Gegensatz dazu streben die Tech-Enthusiasten (Segment 3) nach VR-Endgeräten mit einem möglichst hohen Innovationsgrad. Sie bevorzugen in den simulierten Kaufentscheidungen insbesondere Produkte, die über eine 4K-Auflösung verfügen oder innovative Interaktionsmodalitäten unterstützen. Diese Erkenntnisse sollen Hardwareanbietern, Entwicklern, Verbraucherschützern und Regulatoren dabei helfen, die aktuellen Entwicklungen im VR-Ökosystem besser zu verstehen.

Bei der Interpretation der Ergebnisse dieses Beitrages sind Limitationen zu beachten, die Ansätze für zukünftige Forschungsarbeiten liefern. Erstens hängen die Ergebnisse einer Conjoint-Analyse stark von der Auswahl und Formulierung der Attribute und Ausprägungen ab. So repräsentieren die ausgewählten Attribute nicht alle Attribute eines VR-Headsets, da z. B. Faktoren wie das Sichtfeld oder der Tragekomfort nicht abgefragt wurden. Zukünftige Forschungsarbeiten können untersuchen, welchen Einfluss weitere Attribute auf die Auswahl von VR-Headsets haben. Zweitens kann durch die ungleichmäßige Verteilung der Ausprägungen je Attribut der sog. Number-of-Levels Effekt aufgetreten sein, der die Gewichtung der Attribute verzerrt (Wittink et al. 1992). In zukünftigen Arbeiten sollte die Umfrage aus diesem Grund mit einer gleichverteilten Anzahl von Ausprägungen wiederholt werden. Zudem wurden in der vorliegenden Untersuchung ausschließlich deutsche Studierende befragt. Somit können die Ergebnisse durch das Alter und soziokulturelle Einflüsse verzerrt sein. Es empfiehlt sich daher, die Befragung zukünftig erneut mit einem repräsentativen Sample durchzuführen.

## Anhang

**Tab. 4:** Durchschnittliche Teilnutzenwerte je Marktsegment

Attribut		Teilnutzenwerte			
Ausprägung	Preisjäger (40,89 %)		Datenschützer (22,22 %)	Tech-Enthusiasten (36,89 %)	Gesamte Stichprobe
*Preis*					
400 €	5,199		2,260	3,042	3,750
650 €	0,933		0,558	0,720	0,771
900 €	−6,132		−2,819	−3,762	−4,521
*Interaktion*					
Controller	−2,751		−2,271	−4,641	−3,342
Finger-Tracking	−0,067		0,461	0,571	0,286
Hand-Tracking	2,819		1,811	4,070	3,057
*Displayqualität*					
Niedrig	−2,337		−1,523	−1,971	−2,021
Moderat	−1,178		−1,400	−1,778	−1,449
Hoch	3,515		2,923	3,750	3,470
*Datenschutzbestimmung*					
Facebook	−1,469		−4,087	−1,583	−2,093
Oculus	−1,054		0,181	−0,388	−0,534
Individualisierbar	2,523		3,905	1,971	2,627
*Nutzungsanforderung*					
Stationär	−0,658		−1,403	−0,574	−0,792
Standalone	0,658		1,403	0,574	0,792

## Footnotes

1 Eine Übersicht der durchschnittlichen Teilnutzenwerte je Segment befindet sich in Tab. 4 im Anhang.

2 Wie von Orme (2002) empfohlen, wurden zur Berechnung der relativen Wichtigkeiten je Segment die Durchschnittswerte der relativen Wichtigkeiten aller Mitglieder eines Segmentes gebildet.
